# SnS_2_@C Hollow Nanospheres with Robust Structural Stability as High-Performance Anodes for Sodium Ion Batteries

**DOI:** 10.1007/s40820-019-0243-7

**Published:** 2019-02-21

**Authors:** Shuaihui Li, Zhipeng Zhao, Chuanqi Li, Zhongyi Liu, Dan Li

**Affiliations:** 1 College of Chemistry and Molecular Engineering, Zhengzhou University, Zhengzhou, 450001 Henan People’s Republic of China; 2 Henan Institute of Advanced Technology, Zhengzhou University, Zhengzhou, 450001 Henan People’s Republic of China

**Keywords:** SnS_2_@C, Hollow nanospheres, Anode materials, Sodium ion batteries

## Abstract

Constructing unique and highly stable structures with plenty of electroactive sites in sodium storage materials is a key factor for achieving improved electrochemical properties through favorable sodium ion diffusion kinetics. An SnS_2_@carbon hollow nanospheres (SnS_2_@C) has been designed and fabricated via a facile solvothermal route, followed by an annealing treatment. The SnS_2_@C hybrid possesses an ideal hollow structure, rich active sites, a large electrode/electrolyte interface, a shortened ion transport pathway, and, importantly, a buffer space for volume change, generated from the repeated insertion/extraction of sodium ions. These merits lead to the significant reinforcement of structural integrity during electrochemical reactions and the improvement in sodium storage properties, with a high specific reversible capacity of 626.8 mAh g^−1^ after 200 cycles at a current density of 0.2 A g^−1^ and superior high-rate performance (304.4 mAh g^−1^ at 5 A g^−1^).

**Electronic supplementary material:**

The online version of this article (10.1007/s40820-019-0243-7) contains supplementary material, which is available to authorized users.

## Highlights

- Core–shell structured SnS_2_@C hollow nanospheres were synthesized.
- The uniform carbon coating and hollow structure can alleviate the mechanical strain and therefore electrochemical performance.

**Electronic supplementary material:**

The online version of this article (10.1007/s40820-019-0243-7) contains supplementary material, which is available to authorized users.

## Graphical abstract

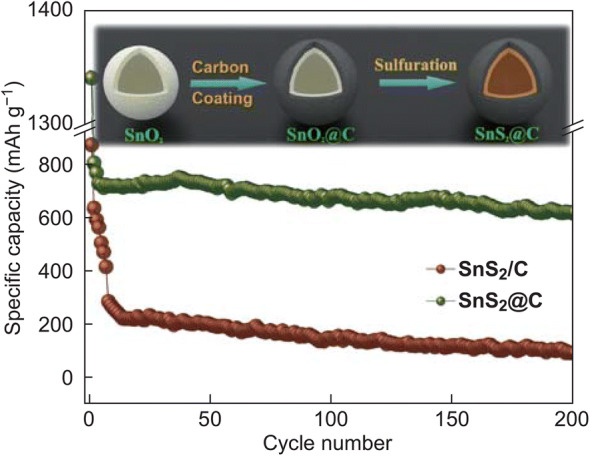

## Introduction

Over-engineering and usage of lithium ion batteries have caused a sudden reduction in lithium sources on earth, along with soaring lithium prices, compared with the previous two decades [1–6]. On the contrary, sodium resources are abundant and even unlimited, accounting for the fourth largest abundance of all elements on a global scale [7]. The advantages of low-cost and similarity in physicochemical properties make sodium ion batteries a promising and reasonable alternative to lithium ion batteries [8–10]. As a result, there is urgency in exploring optimized and high-performance electrode materials for sodium ion batteries [11, 12].

Among the anode materials that have been reportedly applied to sodium ion batteries [13–19], tin and tin-based compounds have attracted intensive attention as sodium storage materials, based on the alloying formation of Na_15_Sn_4_. The high theoretical capacity of 1136 mAh g^−1^ enables SnS_2_ to stand out from the list of tin-based anode materials (847 mAh g^−1^ for metallic tin, 1022 mAh g^−1^ for SnS) because of the additional capacity contribution of the conversion reaction [20]. Like most alloying materials, poor cycling stability is the most serious obstacle of SnS_2_ to achieve decent electrochemical performance, which is due to the greatly destructive volume expansion and extraction during the sodiation and de-sodiation processes, resulting in the loss of contact between electrode materials and current collectors.

Up to now, reported structural designs of SnS_2_ have mostly been focused on two-dimensional (2D) structures as electrode materials for sodium ion batteries [13, 15, 20–29], because of its own Cdl_2_-type hexagonal layer nature [20]. Graphene is regarded as the most favorable substrate to be composited with SnS_2_, considering the structural compatibility of two-layered materials, which act to restrain the volume variation in the electrochemical reactions. For example, Zhang et al. [24] reported a hybrid consisting of few-layered SnS_2_ on reduced graphene oxide nanosheets. Qu et al. [21] integrated SnS_2_ with reduced graphene oxide via a hydrothermal reaction. Liu et al. [13] restacked exfoliated SnS_2_ on graphene sheets by the hydrolysis of lithiated SnS_2_. Tu et al. [28] fabricated a sandwich composite combining few-layered SnS_2_ nanosheets with reduced graphene oxide nanosheets. However, there are few reports on three-dimensional (3D) nanostructured SnS_2_ anode materials for sodium ion batteries, in part owing to the difficulties encountered in the multi-step and time-consuming synthesis procedure or the post-removal of the employed templates or substrates.

Here, a facile template-free hydrothermal reaction followed by an annealing approach has been applied to synthesize 3D SnS_2_@carbon hollow nanospheres (SnS_2_@C). The hollow structure could grant free interior space to accommodate volume expansion and, thus, alleviate mechanical stress. The structure also produces a high surface area to facilitate the presence of more electroactive sites for reactions and facile permeation of electrolyte in electrode materials, as well as a shortened diffusion path for ions and electrons. To reinforce structural stability, a uniform carbon shell was adopted to cover the surface of the hollow nanospheres to suppress the volume variation during electrochemical reactions. Combining the virtues of the core–shell and hollow structures, the as-prepared SnS_2_@C hollow nanospheres exhibited decent cycling performance with a high reversible specific capacity of 626.8 mAh g^−1^ after 200 cycles at a current density of 0.2 A g^−1^, and high-rate capability with a capacity of 304.4 mAh g^−1^ at 5 A g^−1^.

## Experimental

### Sample Preparation

#### Synthesis of SnO_2_ Hollow Nanospheres

SnO_2_ hollow spheres were synthesized by a facile one-pot template-free approach [30]. In a typical synthesis, 0.24 g of urea was added to a mixture of 15 mL of deionized water and 25 mL of ethanol, with stirring for 15 min. Then, 0.195 g of potassium stannate trihydrate (K_2_SnO_3_·3H_2_O) was added to the as-obtained solution, which was vigorously stirred at 25 °C for another 1 h. Afterward, the resultant mixture was transferred to a Teflon-lined stainless steel autoclave and then heated at 220 °C for 24 h in an electric oven. After the reaction, the as-obtained SnO_2_ hollow spheres were centrifugally separated from the suspension and washed with deionized water several times before being dried in a vacuum oven at 70 °C overnight.

#### Synthesis of SnO_2_@C Hollow Nanospheres

First, 150 mg of the as-obtained SnO_2_ hollow nanospheres was dispersed in 100 mL of Tris buffer solution that was stirred for 30 min. Then, 100 mg of dopamine-HCl was added to the solution with vigorous stirring for 12 h. The resultant precipitate was collected by centrifugation and dried at 70 °C for 6 h. Then, the sample was placed in an alumina crucible in the tube furnace, which was heated to 500 °C at a speed of 5 °C min^−1^ under flowing argon gas. After 2 h of reaction, the furnace was left to cool down to 25 °C before the samples were removed from the tube furnace.

#### Synthesis of SnS_2_@C Hollow Nanospheres

The as-prepared SnO_2_@C hollow nanospheres was loaded in an alumina boat, which was placed in a larger, covered alumina boat containing excess thioacetamide outside the inner alumina boat. Then, the alumina boats were transferred to a tube furnace under vacuum and annealed at 300 °C for 2 h at a heating speed of 5 °C min^−1^.

#### Synthesis of SnS_2_/C Bulks

SnS_2_/C bulks were synthesized under identical synthesis conditions as those for SnS_2_@C hollow nanospheres, except, the rapid heating speed was 30 °C min^−1^ during the sulfidation procedure.

#### Synthesis of Bare SnS_2_

Bare SnS_2_ was synthesized under identical synthesis conditions as those for SnS_2_@C hollow nanospheres through the sulfidation of SnO_2_ hollow nanospheres without the carbon coating procedure.

### Material Characterization

The morphologies of samples were characterized by field-emission scanning electron microscopy (FE-SEM, JSM-7500F, JEOL) and transmission electron microscopy (TEM, JEM-2100F, JEOL). X-ray powder diffraction (XRD) was carried out with Cu-K*α* radiation (*λ* = 1.5406 Å) over the range of 2*θ* = 5°–90°. In situ X-ray diffraction (XRD) measurements were performed on a D8 Advance X-ray diffractometer with Cu-K*α* X-ray radiation (*λ* = 1.5406 Å) scanned in the range of 10°–49°. An in situ battery was designed with a Be window for X-ray penetration. The in situ XRD data were recorded upon initial discharging. The (001), (100), (101), and (102) crystal planes of SnS_2_ were chosen for in situ monitoring during the discharge/charge processes at 0.15 A g^−1^. The Raman spectra of the samples were measured using a LabRAM HR Evolution Raman spectrometer. X-ray photoelectron spectroscopy (XPS) experiments were conducted on an ESCALAB 250Xi X-ray photoelectron spectrometer.

### Electrochemical Measurements

Electrochemical measurements were carried out with coin cells, which were assembled in a glove box filled with argon atmosphere. The electrode slurries of the obtained materials were prepared by thoroughly mixing the active material, poly(vinylidene difluoride) (PVDF), and acetylene black in a weight ratio of 8:1:1 in N-methyl pyrrolidone (NMP). The cells were constructed with sodium foil as the anode, prepared active material as the cathode, glass microfiber as the separator, and 1-M NaClO_4_ in a mixture of ethylene carbonate (EC) and propylene carbonate (PC) (1:1 by volume), with 5 wt% fluoroethylene carbonate (FEC) additive as the electrolyte. The average mass of active materials loading per plate was 0.9 mg cm^−1^. The charge/discharge cycling was performed using a battery tester (LANHE CT2001A) with a voltage range between 0.01 and 3.0 V. An electrochemistry workstation (CHI660E) was utilized to conduct measurements in cyclic voltammetry (CV) and electrochemical impedance spectroscopy (EIS). EIS was conducted over the frequency range from 100 kHz to 0.01 Hz at an open circuit voltage. The SnS_2_@C electrodes were first sodiated and then de-sodiated in the cells by discharging to 0.01 V and charging to 3.0 V at a constant current of 0.20 mA g^−1^, respectively. Then, the electrodes were disassembled in a glove box filled with argon atmosphere for ex situ TEM measurements.

## Results and Discussion

**Fig. 1:**
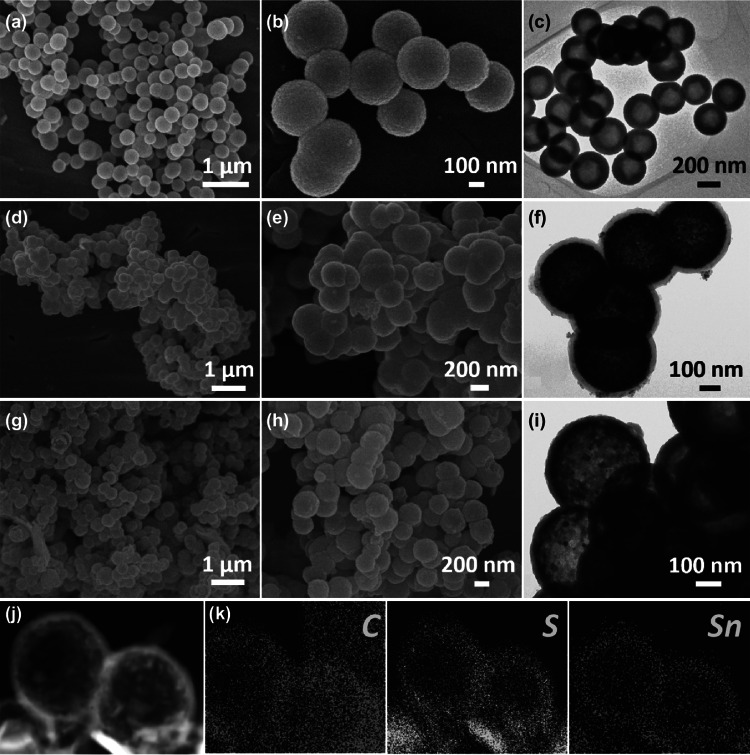
**a**, **b** SEM and **c** TEM images of SnO_2_ hollow nanosphere precursor. **d, e** SEM and **f** TEM images of intermediate SnO_2_@C. **g, h** SEM and **i, j** TEM images of composite SnS_2_@C. **k** element mapping images of carbon, sulfur, and tin

The SnO_2_ precursor was prepared via a facile one-pot template-free approach through the hydrolysis of K_2_SnO_3_·3H_2_O under hydrothermal conditions. The scanning electron microscopy (SEM) images reveal the obtained SnO_2_ to exhibit high monodispersity and a uniform spherical morphology, 250–350 nm in size, as shown in Fig. 1a, b. The distinct contrast difference between the center and edge, shown in the TEM image in Fig. 1c, indicates the hollow feature of the obtained monodispersed SnO_2_ nanospheres with a shell thickness of about 80 nm. After carbon coating by the thermal decomposition of dopamine, the SnO_2_ nanospheres are well encapsulated in the uniform carbon shells, with a thickness of ~ 30 nm, to form intermediate SnO_2_@C. The final product of SnS_2_@C was obtained after sulfidation using thioacetamide as a sulfur source, which inherited the structure and morphology of the SnO_2_@C hollow nanospheres, as shown in Fig. 1g–i. The elemental mapping images in Fig. 1k further indicates the hollow character of SnS_2_@C and the distributions of tin and sulfur in the carbon shells.

**Fig. 2:**
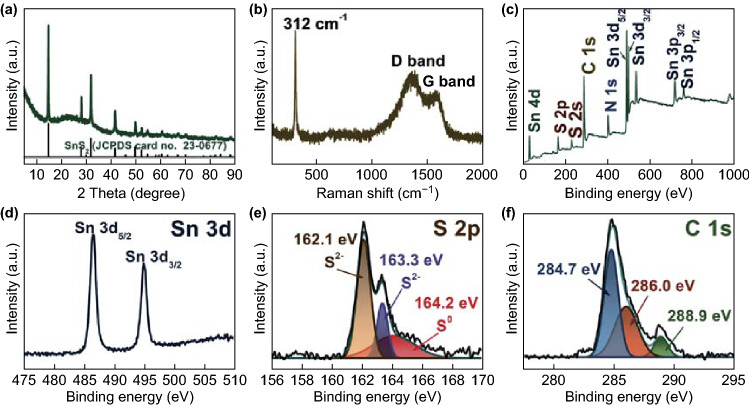
**a** XRD pattern, **b** Raman spectrum of SnS_2_@C, and **c** survey XPS spectrum of SnS_2_@C. Corresponding high-resolution XPS spectra of **d** Sn 3d, **e** S 2p, and **f** C 1s

As evidenced by the X-ray diffraction (XRD) pattern shown in Fig. 2a, all the diffraction peaks are well indexed to the hexagonal phase SnS_2_ (JCPDS card no. 23-0677), indicating the complete sulfidation of SnO_2_@C. No carbon peak can be detected in the XRD pattern, suggesting the low content or amorphous nature of the carbon layer. The carbon content in the SnS_2_@C sample was determined through CHNS elemental analysis, with a value of 17.6 wt%. Considering the XRD result, the thermal decomposition of dopamine generated disordered carbon shells on the hollow spheres, which can be further supported by the Raman spectrum, showing a higher intensity for the disorder-induced D band than that for the graphitic G band. The sharp peak, located at 312 cm^−1^, can be ascribed to in-plane vibrational modes in the S–Sn–S plane of hexagonal SnS_2_ [24]. The surface feature and element states of the SnS_2_@C were validated using XPS. The survey spectrum (Fig. 2c) indicates that the composite is composed of Sn, S, and C elements. The Sn 3d high-resolution XPS spectrum shows two peaks, located at 486.5 and 494.9 eV, corresponding to Sn 3d_5/2_ and Sn 3d_3/2_ of Sn^4+^, respectively [31]. The S 1s peak can be fitted to peaks at 162.1 and 163.3 eV, which can be ascribed to S^2−^ in SnS_2_, and to a peak at 164.2 eV, corresponding to elemental S [11]. For the C 1s spectrum, the XPS peak can be de-convolved into three components of carbon species at 284.7, 286.0, and 288.9 eV, indicating the existence of non-oxygenated carbon, carbon in C–N bonds, and C=N bonds, respectively [24, 32, 33]. The Brunauer–Emmett–Teller surface area of SnS_2_@C was measured to be 93.1 m^2^ g^−1^, which is higher than that of SnS_2_/C, with a value of 75.3 m^2^ g^−1^, as shown in Fig. S12.

**Fig. 3:**
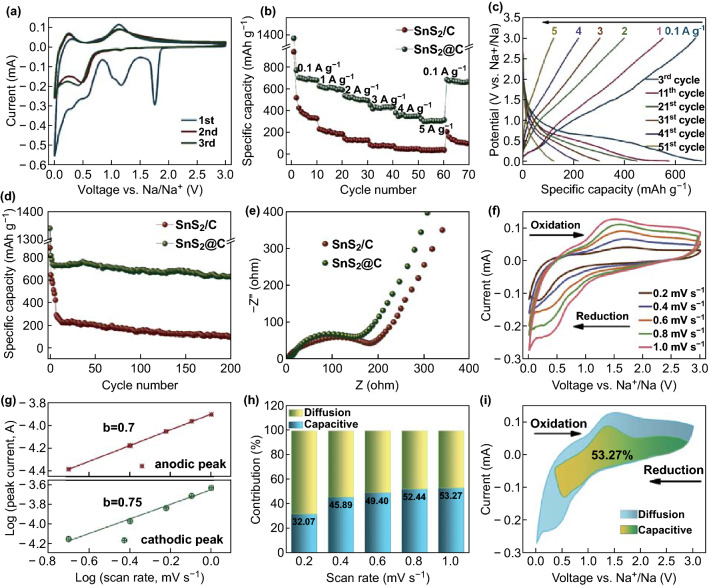
**a** CV curves of SnS_2_@C hollow nanospheres for the first three cycles at a scan rate of 0.1 mV s^−1^. **b** Rate capabilities of SnS_2_/C and SnS_2_@C. **c** Discharge/charge profiles of SnS_2_@C at different current densities (corresponding to **b**). **d** Cycling performance of SnS_2_/C and SnS_2_@C at a current density of 0.2 A g^−1^. **e** Nyquist plots of SnS_2_/C and SnS_2_@C. **f** CV curves of SnS_2_@C at various scan rates. **g** Relationship between log *i* and log *v* plots of anodic and cathodic peaks. **h** Contribution ratios of capacitive capacity of SnS_2_@C at various scan rates. **i** Capacitive contribution and diffusion contribution to the charge storage at a scan rate of 1 mV s^−1^

CV was conducted to evaluate the sodiation/de-sodiation reactions of the SnS_2_@C hollow nanospheres at a scan rate of 0.1 mV s^−1^, as shown in Fig. 3a. The distinct peaks in the initial cathodic scan, located at 1.76 and 1.18 V, can be attributed to the Na^+^ intercalation into SnS_2_ to form Na_*x*_SnS_2_ [13, 32]. The broad peak, ranging from 0.59 to 0.3 V, is related to the conversion reaction of Na_*x*_SnS_2_ to form Na_2_S and metallic Sn and, subsequently, the alloying reaction between the formed Sn and Na^+^ as well as the formation of solid electrolyte interphase film. In the anodic scan, the peak at 0.25 V corresponds to the extraction of Na^+^ from Na_*x*_Sn. Meanwhile, the peak at 1.14 V can be assigned to the reformation of Na_*x*_SnS_2_ [34].

To elucidate the structural virtue, SnS_2_/C bulks were prepared to provide a comparison under the identical synthesis conditions as those for SnS_2_@C hollow nanospheres except a rapid heating rate of 30 °C min^−1^ in the sulfidation procedure. The carbon content of SnS_2_/C bulks is 15.6 wt% by CHNS elemental analysis. Figure 3b presents the comparison of the rate capabilities of SnS_2_@C hollow nanospheres and SnS_2_/C bulks. The average specific capacities of SnS_2_@C are 695.5, 604.1, 507.6, 427.2, and 350.8 mAh g^−1^ at current densities of 0.1, 1, 2, 3, and 4 A g^−1^, respectively. Remarkably, SnS_2_@C delivered a high capacity of 304.4 mAh g^−1^ at a relatively large current density of 5 A g^−1^. When the current density returned to 0.1 A g^−1^, the specific capacity recovered to 665.5 mAh g^−1^, showing good reversibility. By contrast, the SnS_2_/C shows lower capacities at various current densities (only 34.8 mAh g^−1^ at 5 A g^−1^). Figure 3c presents the representative discharge/charge profiles of SnS_2_@C under different current densities, corresponding to Fig. 3b. In terms of cyclic stability, SnS_2_@C exhibits superior capacity retention compared with SnS_2_/C, delivering a specific capacity of 626.8 mAh g^−1^ at the 200th cycle under 0.2 A g^−1^, which is much larger than that of SnS_2_/C bulks with a value of 110.5 mAh g^−1^, as shown in Fig. 3d. EIS measurements were conducted to reveal the difference in electrochemical behavior between SnS_2_/C and SnS_2_@C. The Nyquist plots and resistance values simulated from modeling the experimental impedance spectra are shown in Figs. 3e, S14, and Table S1, which show the lower charge transfer resistance of SnS_2_@C hollow nanospheres as compared with that of SnS_2_/C bulks.

To clarify the reaction kinetics underlying the superior sodium storage of SnS_2_@C, CV tests at various scan rates of 0.2, 0.4, 0.6, 0.8, and 1 mV s^−1^ were conducted to investigate the sodiation and de-sodiation processes. As shown in Fig. 3f, the cathodic and anodic peak current responses increase with increasing scan rate, with a gradual broadening of the peaks. The relationship between the peak current and scan rate was calculated based on the equation of log *i* = *b*·log*v* + log *a*, where *a* and *b* are two variables, and the *b*-value determines the sodiation and de-sodiation types, which is between 0.5 and 1.0. The *b*-value approaching 1.0 refers to a capacitive-dominated process, while *b*-values close to 0.5 indicate that the electrochemical reactions are under the control of ion diffusion. The *b*-values are calculated to be 0.7 and 0.75 for the anodic and cathodic peaks, respectively, indicating a combined effect between capacitive and ion diffusion processes on sodium storage. The capacitive contribution can be further quantitatively determined based on the equation *i* = *k*_1_·*v* + *k*_2_·*v*^1/2^, which includes the capacitive effect (*k*_1_·*v*) and diffusion-controlled reaction (*k*_2_·*v*^1/2^). The capacitive contributions are calculated to be 32.07%, 45.89%, 49.40%, and 52.44% at the scan rates of 0.2, 0.4, 0.6, and 0.8 mV s^−1^, respectively, as shown in Fig. 3h. Compared with SnS_2_/C, the large values of the capacitive contribution ratios of SnS_2_@C can be attributed to the high surface area stemming from the hollow structure. Figure 3i displays the ratio of the capacitive contribution to the total capacity for the SnS_2_@C hollow nanospheres, with a value of 53.27% at a scan rate of 1.0 mV s^−1^.

Compared with the SnS_2_/C bulks and reported tin sulfides materials, 3D SnS_2_@C hollow nanospheres exhibit outstanding electrochemical performance compared with 0D SnS_2_/C composites, 2D SnS_2_/graphene composites, and SnS_2_-based hybrids, as anode materials for sodium ion batteries, as summarized in Table S2 in Supporting Information. This performance could be attributed to the following characteristics: (1) the hollow structure facilitates high surface permeability because of the large electrolyte/electrode interfaces and more reactive sites for sodium ions. More importantly, the inner hollow space can accommodate the large volume expansion (324%) and, therefore, absorb the stress generated in the reaction processes; (2) the uniform carbon shells efficiently buffer the volume variation during the sodiation and de-sodiation processes, maintaining the mechanical stability and structural integrity of SnS_2_ hollow nanospheres. Moreover, the connected carbon shells provide a conductive framework to promote electron transfer and enhance the electrical conductivity of the composite; (3) the annealed hollow structure and large surface area are favorable to the capacitive contribution during the charge and discharge processes, producing a preferred effect on rate capability.

**Fig. 4:**
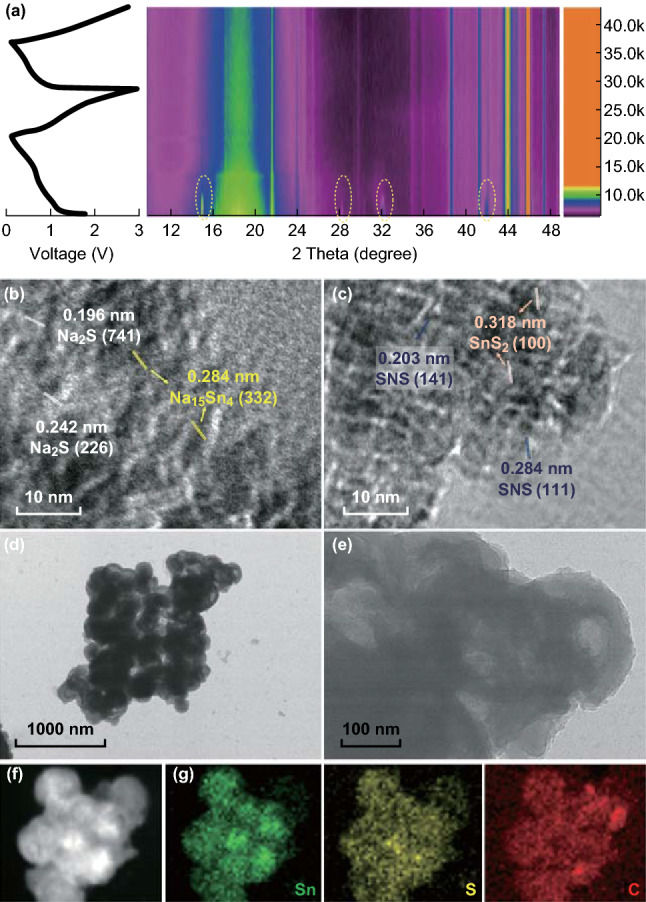
**a** 2D view of in situ XRD patterns during initial galvanostatic discharge and charge of SnS_2_@C hollow nanospheres, HRTEM images at **b** discharge to 0.01 V, and **c** charge to 3.0 V. **d–f** TEM images of SnS_2_@C hollow nanosphere electrode after 100 cycles at 0.5 A g^−1^. **g** Corresponding element mapping images of Sn, S, and C

To uncover the underlying reaction mechanism of SnS_2_@C hollow nanospheres, a combined study of in situ XRD and HRTEM images was conducted for further analysis, as shown in Fig. 4a–c. The 2D view of in situ XRD patterns displays the gradual disappearance of the main peaks of SnS_2_ at 15.3°, 28.2°, 32.1° and 41.9° during the initial discharge process, indicating the participation of SnS_2_ by Na^+^ intercalation. As the reaction progresses, no new peaks, corresponding to Na_2_S nor Na_*x*_Sn, can be discerned in the XRD patterns, which may be due to the low crystallinity [20]. Meanwhile, the HRTEM image (Fig. 4b) provides clear evidence of the existence of Na_2_S and sodiated Sn, Na_15_Sn_4_, at the fully discharged state, verifying the occurrence of the alloying reaction between Na^+^ and the formed Sn. When de-sodiation proceeds to 3.0 V (Fig. 4c), the detection of SnS and SnS_2_ demonstrates the partial irreversible recovery of SnS_2_ from metallic Sn, which can be ascribed to sluggish reaction kinetics [20]. Figure 4d–f shows the TEM images of the SnS_2_@C electrode after 100 cycles at the current density of 0.5 A g^−1^, demonstrating the structural stability. It is found that SnS_2_@C hollow nanospheres are amorphous to some extent, and the structural integrity is well maintained after cycling.

## Conclusion

In summary, SnS_2_@C hollow nanospheres were synthesized via a facile solvothermal route, followed by an annealing treatment. Standing out from the various widely reported 2D structured SnS_2_ electrode materials for sodium ion batteries, the obtained SnS_2_@C hollow nanospheres possess multiple merits, including many active sites, high surface permeability, desirable void space for volume expansion, and favorable kinetics by the virtue of a high face-to-volume ratio. Combined with the effective buffering effects of the carbon coating strategy, the mechanical stability is considerably improved to withstand repeated charging/discharge processes, showing decent sodium storage performance. The resultant SnS_2_@C hollow nanospheres exhibit high specific capacity and superior rate capability, in part owing to the capacitive contribution for fast sodiation/de-sodiation reaction kinetics, based on the quantitative capacitive analysis. The facile synthesis and satisfied electrochemical properties enable SnS_2_@C hollow nanospheres to be a promising high-performance electrode material for energy storage and conversion.

## Electronic supplementary material

Below is the link to the electronic supplementary material.

Supplementary material 1 (PDF 1445 kb)
