# Supplementary material for: SnS2@C Hollow Nanospheres with Robust Structural Stability as High-Performance Anodes for Sodium Ion Batteries
Source: Nanomicro Lett. 2019 Feb 21;11:14. doi: 10.1007/s40820-019-0243-7 (PMC7770728; doi:10.1007/s40820-019-0243-7)
Supplement: Supplementary file 1 — Supplementary material 1 (PDF 1445 kb) [file 40820_2019_243_MOESM1_ESM.pdf]

Supporting Information for

SnS_2@C Hollow Nanospheres with Robust Structural Stability as High-Performance Anodes for Sodium Ion Batteries

Shuaihui Li[1, 2,] †, Zhipeng Zhao[1, 2,] †, Chuanqi Li[1, 2], Zhongyi Liu[1, 2,] *, Dan Li[1, 2,] *

[1]College of Chemistry and Molecular Engineering, Zhengzhou University, Zhengzhou, Henan Province~~,~~ 450001, People's Republic of China

[2]Henan Institute of Advanced Technology, Zhengzhou University, Zhengzhou, Henan Province~~,~~ 450001, People's Republic of China

†These authors have contributed equally

*Corresponding authors. E-mail: liuzhongyi@zzu.edu.cn (Zhongyi Liu); danli@zzu.edu.cn (Dan Li)

Supplementary Figures and Tables

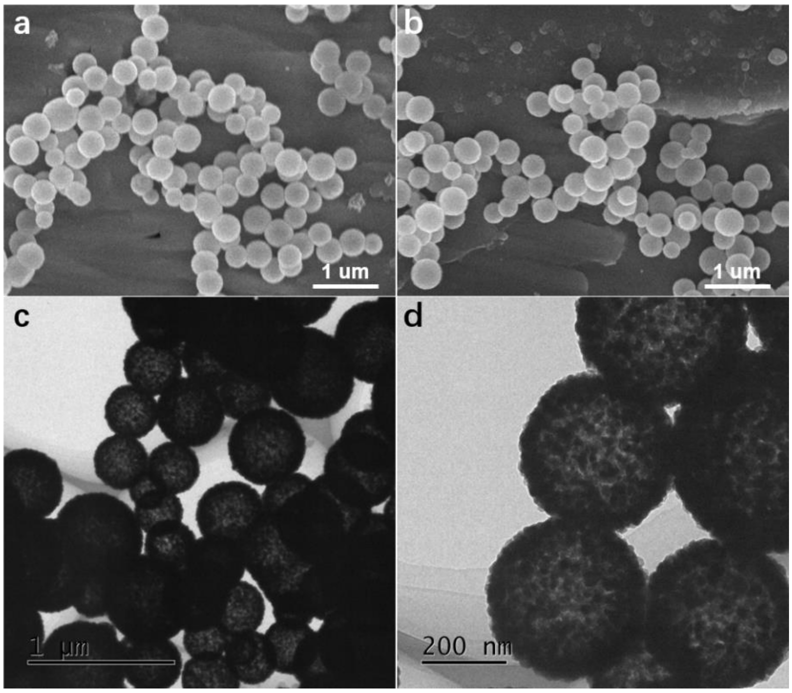

Fig. S1 a, b SEM and **c, d** TEM images of SnO_2 hollow nanospheres precursor

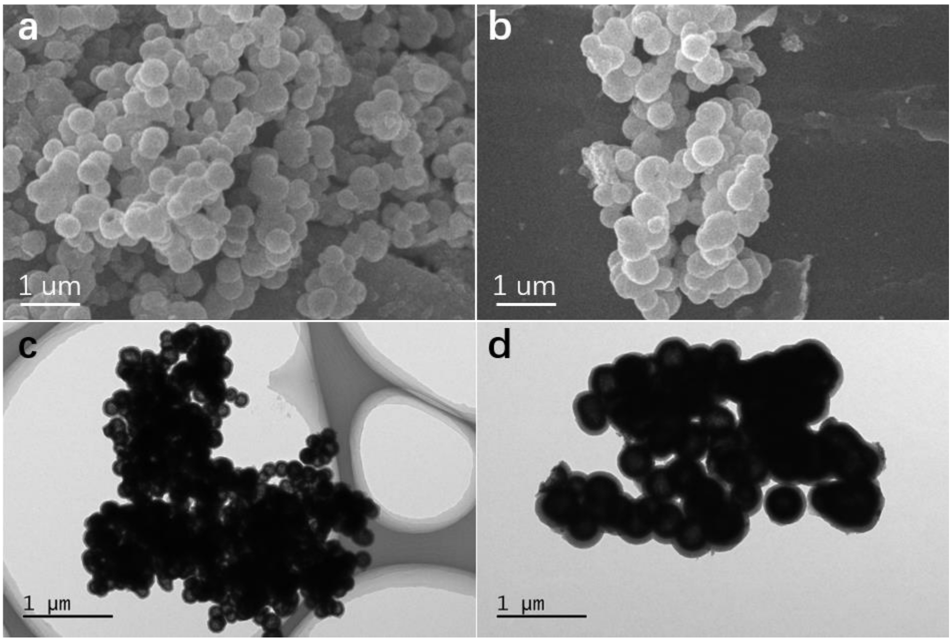

Fig. S2 a, b SEM and **c, d** TEM images of SnO_2@C hollow nanospheres intermediate

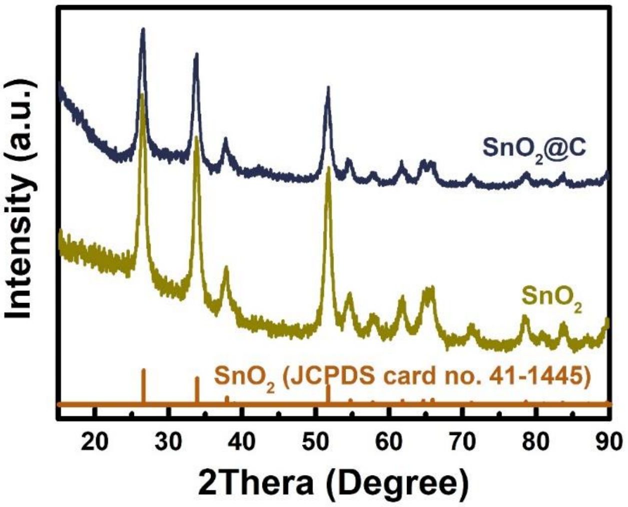

Fig. S3 XRD patterns of SnO_2 and SnO_2@C

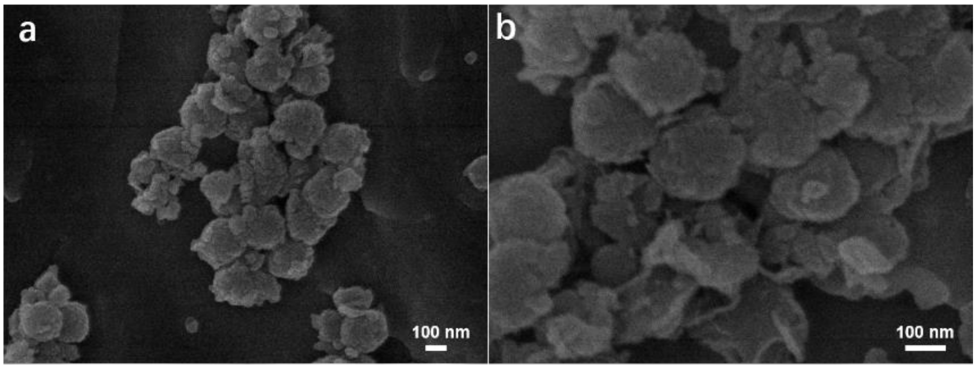

Fig. S4 a, b SEM images of the SnS_2@C obtained at the sulfidation temperature of 320 °C

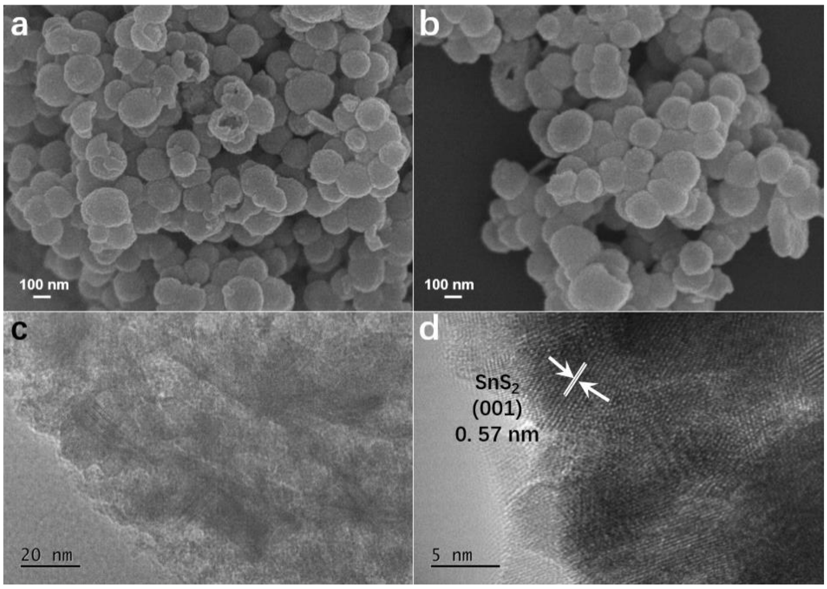

Fig. S5 a, b SEM and **c, d** HRTEM images of SnS_2@C hollow nanospheres

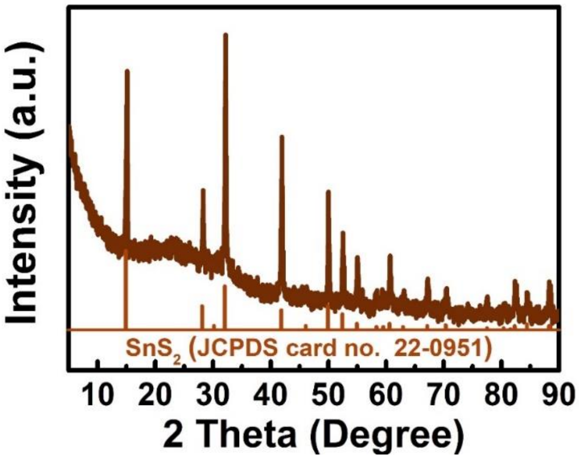

Fig. S6 XRD pattern of SnS_2/C bulks

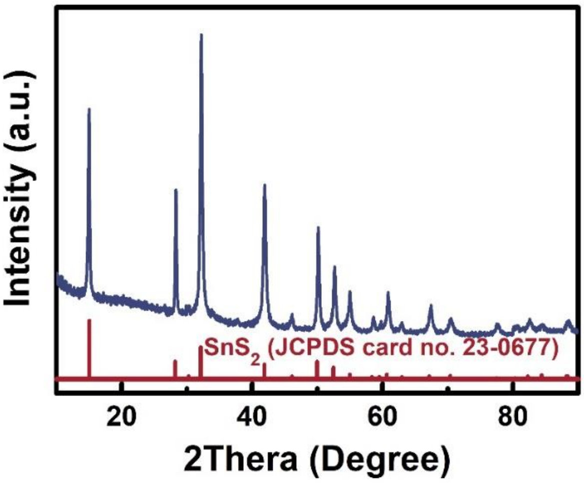

Fig. S7 XRD pattern of the bare SnS_2

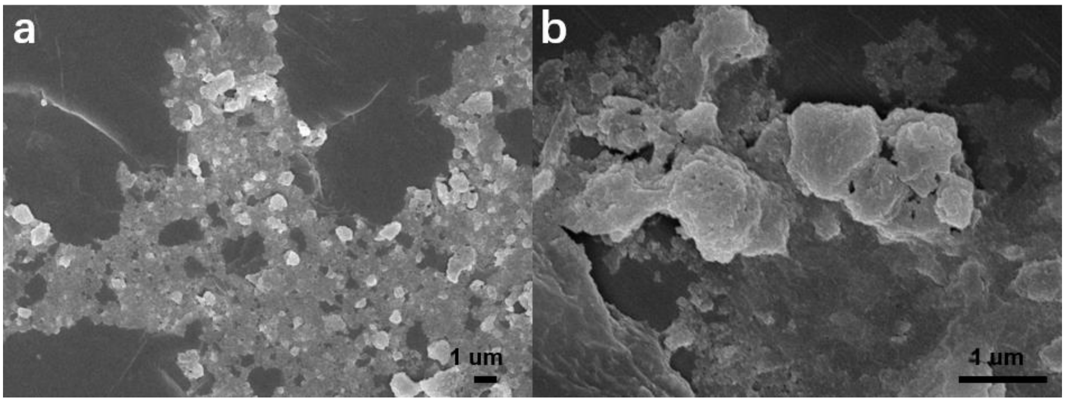

Fig. S8 a, b SEM of the bare SnS_2

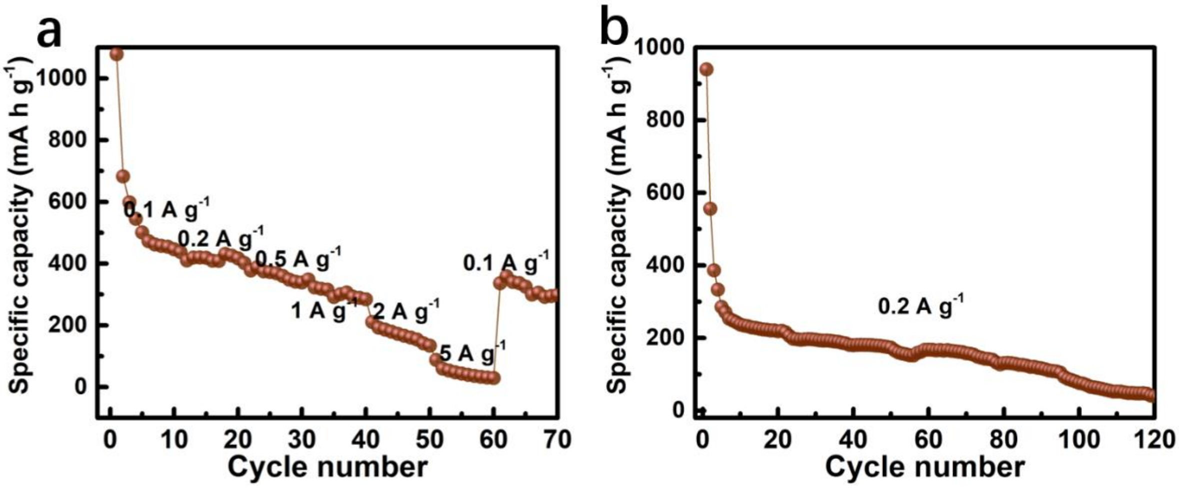

Fig. S9 a Rate capability and **b** cycling performance of the bare SnS_2

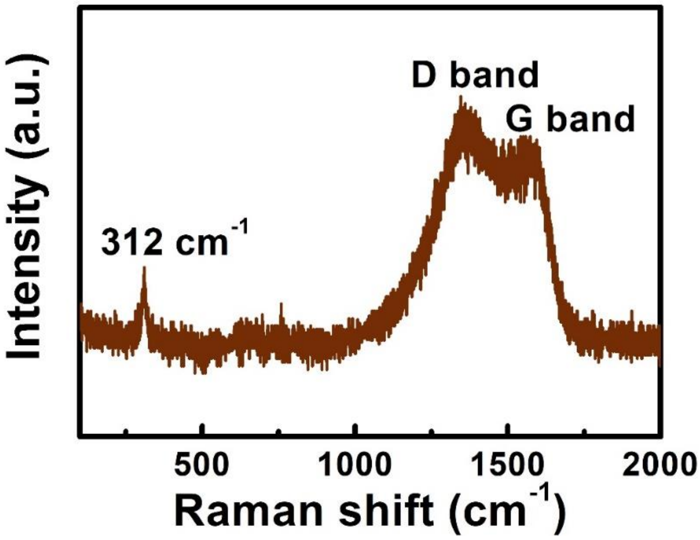

Fig. S10. Raman spectrum of SnS_2/C bulks

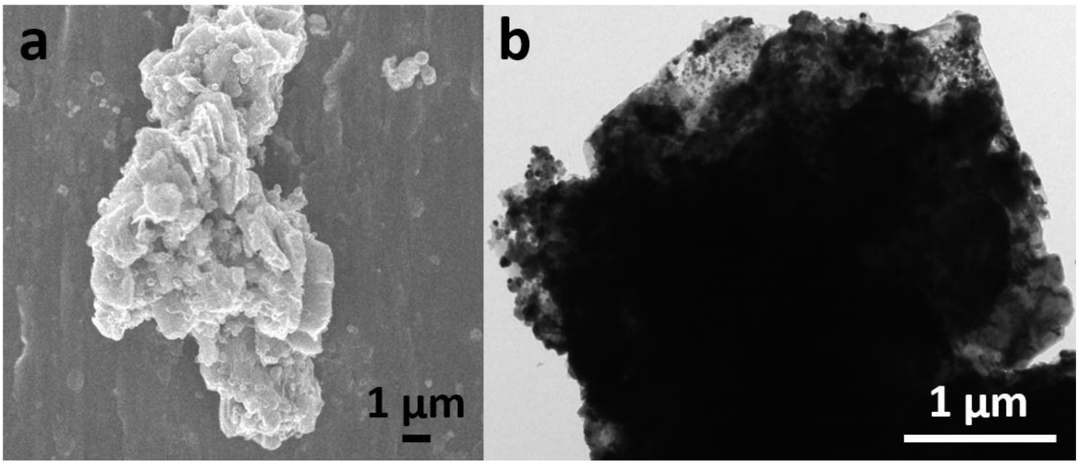

Fig. S11 **a** SEM and **b** TEM images of SnS_2/C bulks

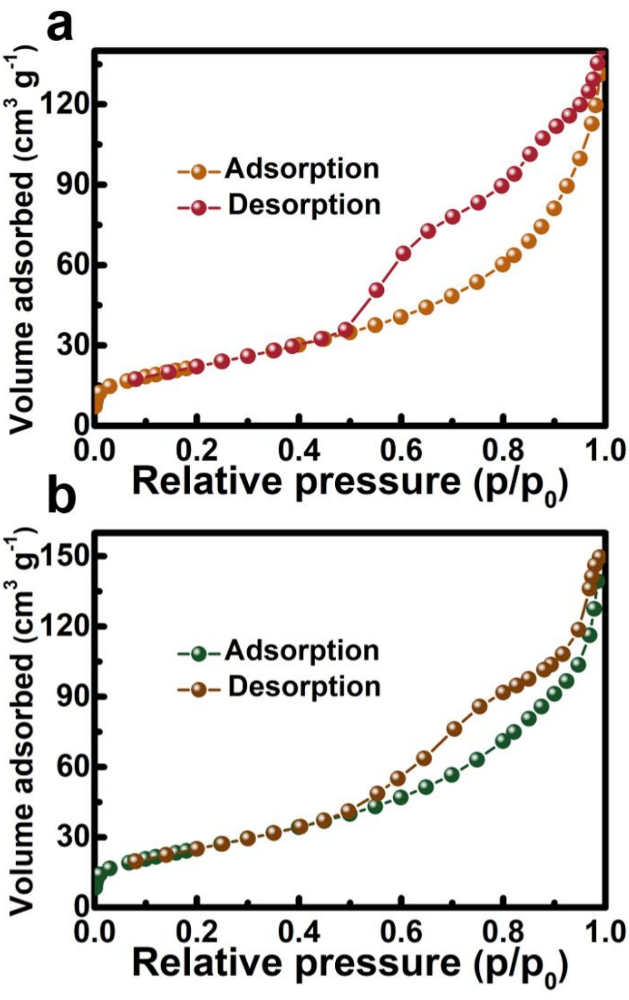

Fig. S12 N_2 adsorption/desorption isotherms of the **a** SnS_2/C bulks and **b** SnS_2@C hollow nanospheres

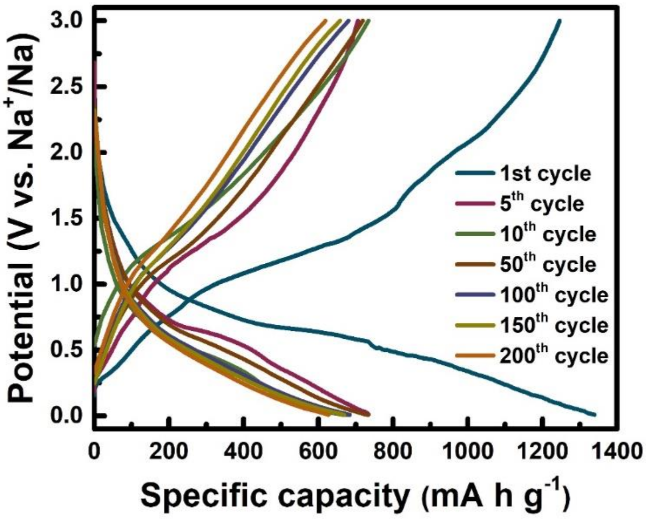

Fig. S13 The charge/discharge profiles of SnS_2@C at different cycles at a current density of 0.2 A g^{-1}

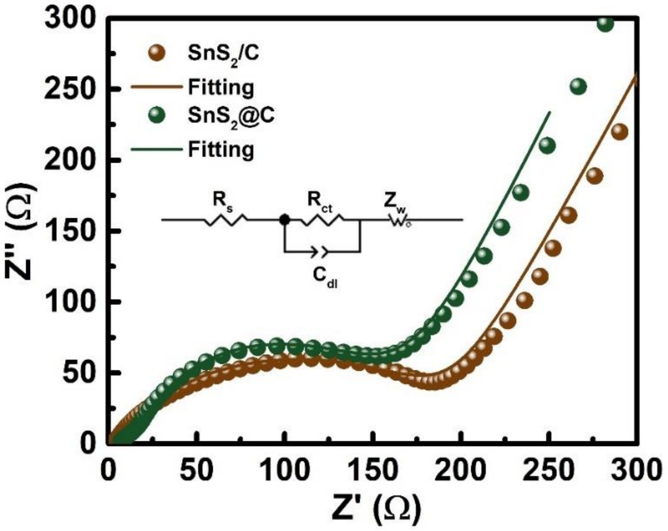

Fig. 14 Experimental (dot) and simulated (line) Nyquist plots of SnO_2/C and SnO_2@C. Inset depicts the equivalent circuit

Table S1 Resistance values simulated from modeling the experimental impedance spectra

Sample	R_s (Ω)	R_{ct} (Ω)
**SnO_2/C**	2.79	189.1
**SnO_2@C**	3.91	161.7

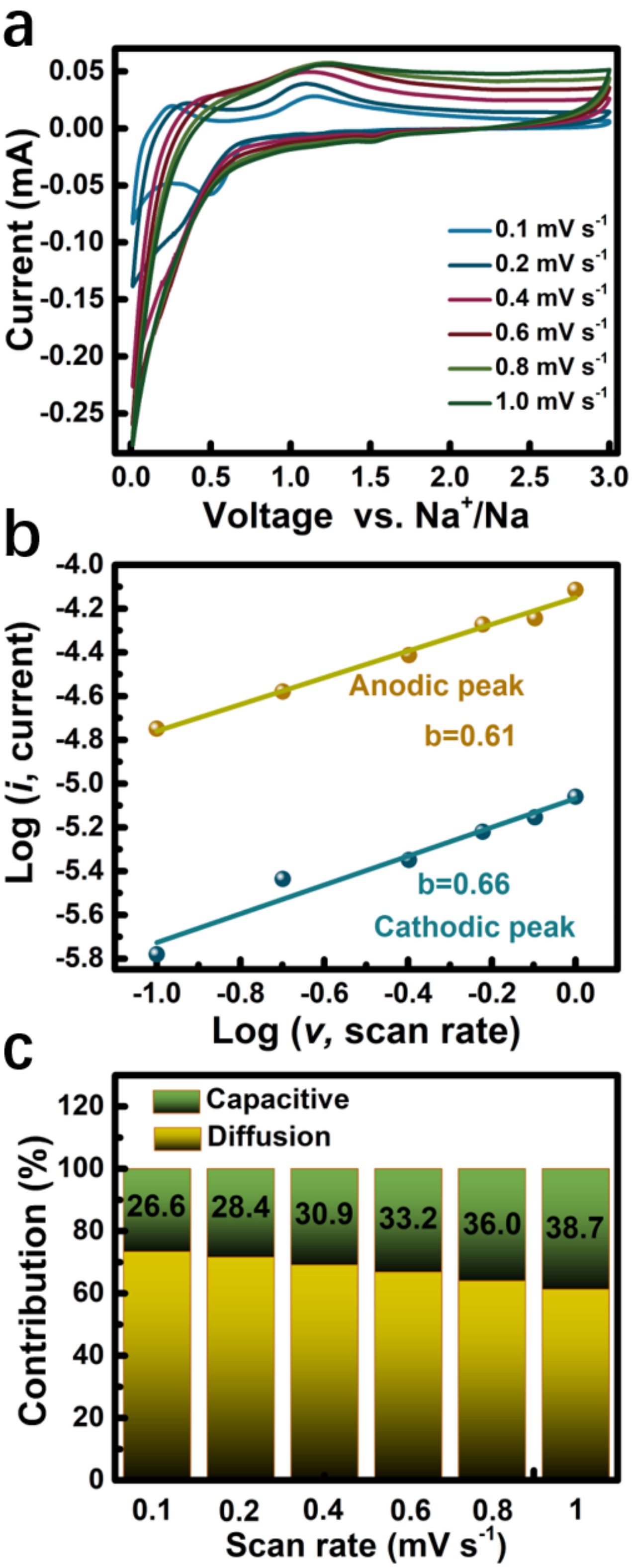

Fig. S15 **a** CV curves at various scan rates, **b** relationship between log *i* and log *v* plots of anodic and cathodic peaks, **c** contribution ratios of capacitive capacity of SnS_2/C at various scan rates

Table S2 Summary of the electrochemical performance of SnS_2-based materials

Structures	Materials	Cycling performance ($mAh\ g^{-1}$)	Rate Capability ($mAh\ g^{-1}$)	Refs.
**0D**	N, S-doped graphene aerogel/SnS_2 nanocrystal	527 after 50 cycles at 0.02 A g^{-1}	340 at 0.8 A g^{-1}	[1]
	Ultrafine SnS_2 nanocrystals/rGO	418 after 100 cycles at 1 A g^{-1}	260 at 10 A g^{-1}	[2]
	SnS_2 ultrafine nanocrystals/graphene	680 after 100 cycles at 0.2 A g^{-1}	250 at 11.2 A g^{-1}	[3]
	SnS_2-rGO composite	450 after 150 cycles at 0.5 A g^{-1}	340 at 2 A g^{-1}	[4]
**2D**	2D SnS_2 nanosheets	647 after 50 cycles at 0.1 A g^{-1}	435 at at 2 A g^{-1}	[5]
	SnS_2@graphene	~520 after 50 cycles at 0.03 A g^{-1}	300 at 7.29 A g^{-1}	[6]
	SnS_2-rGO composite	628 after 100 cycles at 0.2 A g^{-1}	544 at 2 A g^{-1}	[7]
	SnS_2/graphene	650 after 100 cycles at 0.2 A g^{-1}	326 at 4 A g^{-1}	[8]
	2D SnS_2 nanoarray	NA	400 at 10 A g^{-1}	[9]
	Few-layered SnS_2/rGO	509 after 300 cycles at 0.2 A g^{-1}	337 at 12.8 A g^{-1}	[10]
	Few-layered pseudocapacitive SnS_2	338 after 150 cycles at 2.5 A g^{-1}	172 at 12 A g^{-1}	[11]
	SnS_2 nanoplatelet@graphene	670 after 60 cycles at 0.02 A g^{-1}	152 at 0.64 A g^{-1}	[12]
	2D MXene/SnS_2 composites	322 after 200 cycles at 0.1 A g^{-1}	78 at 2 A g^{-1}	[13]
	2D SnS_2/CNTs hybrid	476.3 after 100 cycles at 0.05 A g^{-1}	265.5 at 3.2 A g^{-1}	[14]
	SnS_2@graphene nanosheet arrays	378 after 200 cycles at 1.2 A g^{-1}	348 at 3 A g^{-1}	[15]
	SnS_2 nanowall arrays	576 after 100 cycles at 0.5 A g^{-1}	~370 at 5 A g^{-1}	[16]
	SnS_2/graphene nanocomposites	615.2 after 100 cycles at 0.2 A g^{-1}	501.5 at 2 A g^{-1}	[17]
	SnS_2 nanosheet assemblies	~420 after 100 cycles at 0.5 A g^{-1}	500 at 5 A g^{-1}	[18]
	SnS_2/rGO sandwich hybrid	843 after 100 cycles at 0.1 A g^{-1}	335 at 8.4 A g^{-1}	[19]
	SnS_2 nanoplates	241.5 after 50 cycles at 0.1 A g^{-1}	77 at 5 A g^{-1}	[20]
	SnS_2/S-doped graphene	~300 after 500 cycles at 2 A g^{-1}	150 at 5 A g^{-1}	[21]
	Layered SnS_2 cross-	716.2 after 100 cycles at 0.1 A g^{-1}	445 at 5 A g^{-1}	[22]

	linked/CNTs			
	SnS_2 nanosheets	414 after 50 cycles at 0.05 A g^{-1}	299 at 0.5 A g^{-1}	[23]
**3D**	SnS_2/C nanospheres	570 after 100 cycles at 0.05 A g^{-1}	360 at at 1 A g^{-1}	[24]
	3D SnS_2 flowers/CNT	460 after 20 cycles at 0.02 A g^{-1}	180 at 1.28 A g^{-1}	[25]
	Flower-like SnS_2@rGO	509 after 50 cycles at 0.1 A g^{-1}	102 at 0.4 A g^{-1}	[26]
**Hybrids**	SnS_2/Sb_2S_3 heterostructures/rGO	642 after 100 cycles at 0.2 A g^{-1}	567 at 4 A g^{-1}	[27]
	Hollow SnO_2/SnS_2 hybrids	485.6 after 100 cycles at 0.3 A g^{-1}	245.4 at 2.5 A g^{-1}	[28]
	MoS_2@SnS_2 nanoflakes/graphene	100 after 50 cycles at 0.08 A g^{-1}	145 at 0.32 A g^{-1}	[29]
	SnO_2/SnS_2/CNTs composite	355 after 100 cycles at 0.05 A g^{-1}	105 at 3.2 A g^{-1}	[30]
**Hollow nanospheres**	SnS_2@C hollow nanospheres	626.8 after 200 cycles at 0.2 A g^{-1}	304.4 at 5 A g^{-1}	**This work**

Supplementary References

[1] L. Fan, X. Li, X. Song, N. Hu, D. Xiong, A. Koo, X. Sun, Promising dual-doped graphene aerogel/SnS_2 nanocrystal building high performance sodium ion batteries. ACS Appl. Mater. Interfaces **10**(3), 2637-2648 (2018). https://doi.org/10.1021/acsami.7b18195

[2] Y. Liu, Y. Yang, X. Wang, Y. Dong, Y. Tang, Z. Yu, Z. Zhao, J. Qiu, Flexible paper-like free-standing electrodes by anchoring ultrafine SnS_2 nanocrystals on graphene nanoribbons for high-performance sodium ion batteries. ACS Appl. Mater. Interfaces **9**(18), 15484-15491 (2017). https://doi.org/10.1021/acsami.7b02394

[3] Y. Jiang, M. Wei, J. Feng, Y. Ma, S. Xiong, Enhancing the cycling stability of Na-ion batteries by bonding SnS_2 ultrafine nanocrystals on amino-functionalized graphene hybrid nanosheets. Energ. Environ. Sci. **9**(4), 1430-1438 (2016). https://doi.org/10.1039/C5EE03262H

[4] P.V. Prikhodchenko, D.Y.W. Yu, S.K. Batabyal, V. Uvarov, J. Gun, S. Sladkevich, A.A. Mikhaylov, A.G. Medvedev, O. Lev, Nanocrystalline tin disulfide coating of reduced graphene oxide produced by the peroxostannate deposition route for sodium ion battery anodes. J. Mater. Chem. A **2**(22), 8431-8437 (2014). https://doi.org/10.1039/c3ta15248k

[5] W. Sun, X. Rui, D. Yang, Z. Sun, B. Li et al., Two-dimensional tin disulfide nanosheets for enhanced sodium storage. ACS Nano **9**(11), 11371-11381 (2015).

https://doi.org/10.1021/acsnano.5b05229

[6] T. Zhou, W. K. Pang, C. Zhang, J. Yang, Z. Chen, H.K. Liu, Z. Guo, Enhanced sodium-ion battery performance by structural phase transition from two-dimensional hexagonal-SnS_2 to orthorhombic-SnS. ACS Nano **8**(8), 8323-8333 (2014). https://doi.org/10.1021/nn503582c

[7] B. Qu, C. Ma, G. Ji, C. Xu, J. Xu, Y.S. Meng, T. Wang, J.Y. Lee, Layered SnS_2-reduced graphene oxide composite – a high-capacity, high-rate, and long-cycle life sodium-ion battery anode material. Adv. Mater. **26**(23), 3854-3859 (2014). https://doi.org/10.1002/adma.201306314

[8] Y. Liu, H. Kang, L. Jiao, C. Chen, K. Cao, Y. Wang, H. Yuan, Exfoliated- SnS_2 restacked on graphene as a high-capacity, high-rate, and long-cycle life anode for sodium ion batteries. Nanoscale **7**(4), 1325-1332 (2015). https://doi.org/10.1039/C4NR05106H

[9] D. Chao, P. Liang, Z. Chen, L. Bai, H. Shen et al., Pseudocapacitive Na-ion storage boosts high rate and areal capacity of self-branched 2D layered metal chalcogenide nanoarrays. ACS Nano **10**(11), 10211-10219 (2016). https://doi.org/10.1021/acsnano.6b05566

[10] Y. Zhang, P. Zhu, L. Huang, J. Xie, S. Zhang, G. Cao, X. Zhao, Few-layered SnS_2 on few-layered reduced graphene oxide as Na-ion battery anode with ultralong cycle life and superior rate capability. Adv. Funct. Mater. 2**5**(3), 481-489 (2015). https://doi.org/10.1002/adfm.201402833

[11] R. Thangavel, A. Samuthira Pandian, H. V. Ramasamy, Y.-S. Lee, Rapidly synthesized, few-layered pseudocapacitive SnS_2 anode for high-power sodium ion batteries. ACS Appl. Mater. Interfaces **9**(46), 40187-40196 (2017). https://doi.org/10.1021/acsami.7b11040

[12] X. Xie, D. Su, S. Chen, J. Zhang, S. Dou, G. Wang, SnS_2 nanoplatelet@graphene nanocomposites as high-capacity anode materials for sodium-ion batteries. Chem. –Asian J. **9**(6), 1611-1617 (2014). https://doi.org/10.1002/asia.201400018

[13] Y. Wu, P. Nie, L. Wu, H. Dou, X. Zhang, 2D Mxene/SnS_2 composites as high-performance anodes for sodium ion batteries. Chem. Eng. J. **334**, 932-938 (2018). https://doi.org/10.1016/j.cej.2017.10.007

[14] S. Zhang, H. Zhao, M. Wu, L. Yue, J. Mi, One-pot solvothermal synthesis 2D SnS_2/CNTs hybrid as a superior anode material for sodium-ion batteries. J. Alloys Compd. **737**, 92-98 (2018). https://doi.org/10.1016/j.jallcom.2017.11.389

[15] W. Xu, K. Zhao, L. Zhang, Z. Xie, Z. Cai, Y. Wang, SnS_2@graphene nanosheet arrays grown on carbon cloth as freestanding binder-free flexible anodes for advanced sodium batteries. J. Alloys Compd. **654**, 357-362 (2016). https://doi.org/10.1016/j.jallcom.2015.09.050

[16] P. Zhou, X. Wang, W. Guan, D. Zhang, L. Fang, Y. Jiang, SnS_2 nanowall arrays toward high-performance sodium storage. ACS Appl. Mater. Interfaces **9**(8), 6979-6987 (2017). https://doi.org/10.1021/acsami.6b13613

[17] J. Ye, L. Qi, B. Liu, C. Xu, Facile preparation of hexagonal tin sulfide nanoplates anchored on graphene nanosheets for highly efficient sodium storage. J. Colloid. Interf. Sci. **513,** 188-197 (2017). https://doi.org/10.1016/j.jcis.2017.11.025

[18] Y. Wang, J. Zhou, J. Wu, F. Chen, P. Li et al., Engineering SnS_2 nanosheet assemblies for enhanced electrochemical lithium and sodium ion storage. J. Mater. Chem. A **5**(48), 25618-25624 (2017). https://doi.org/10.1039/C7TA08056E

[19] F. Tu, X. Xu, P. Wang, L. Si, X. Zhou, J. Bao, A few-layer SnS_2/reduced graphene oxide sandwich hybrid for efficient sodium storage. J. Phys. Chem. C **121**(6), 3261-3269 (2017). https://doi.org/10.1021/acs.jpcc.6b12692

[20] Y. Xie, M. Fan, T. Shen, Q. Liu, Y. Chen, SnS_2 nanoplates as stable anodes for sodium ion and lithium ion batteries. Mater. Technol. **31**(11), 646-652 (2016). https://doi.org/10.1080/10667857.2016.1208451

[21] P. Zheng, Z. Dai, Y. Zhang, K.N. Dinh, Y. Zheng et al., Scalable synthesis of SnS_2/S-doped graphene composites for superior Li/Na-ion batteries. Nanoscale **9**(39), 14820-14825 (2017). https://doi.org/10.1039/C7NR06044K

[22] H. Li, M. Zhou, W. Li, K. Wang, S. Cheng, K. Jiang, Layered SnS_2 cross-linked by carbon nanotubes as a high performance anode for sodium ion batteries. RSC Adv. **6**(42), 35197-35202 (2016). https://doi.org/10.1039/C6RA04941A

[23] J. Zhao, X. Yu, Z. Gao, W. Zhao, R. Xu, Y. Liu, H. Shen, One step synthesis of SnS_2 nanosheets assembled hierarchical tubular structures using metal chelate nanowires asa soluble template for improved Na-ion storage. Chem. Eng. J. **332**, 548-555 (2018). https://doi.org/10.1016/j.cej.2017.09.110

[24] J. Wang, C. Luo, J. Mao, Y. Zhu, X. Fan, T. Gao, A.C. Mignerey, C. Wang, Solid-state fabrication of SnS_2/C nanospheres for high-performance sodium ion battery anode. ACS Appl. Mater. Interfaces **7**(21), 11476 (2015). https://doi.org/10.1021/acsami.5b02413

[25] Y. Ren, J. Wang, X. Huang, J. Ding, Three-dimensional SnS_2 flowers/carbon nanotubes network: Extraordinary rate capacity for sodium-ion battery. Mater. Lett. **186**(Supplement C), 57-61 (2017). https://doi.org/10.1016/j.matlet.2016.09.089

[26] Z. Yu, X. Li, B. Yan, D. Xiong, M. Yang, D. Li, Rational design of flower-like tin sulfide @ reduced graphene oxide for high performance sodium ion batteries. Mater. Res. Bull. **96**, 516-523 (2017). https://doi.org/10.1016/j.materresbull.2017.04.048

[27] S. Wang, S. Liu, X. Li, C. Li, R. Zang, Z. Man, P. Li, Y. Wu, G. Wang, SnS_2/Sb_2S_3 heterostructures anchored on reduced graphene oxide nanosheets with superior rate capability for sodium-ion batteries. Chem. - Eur J. **24**, 3873-3881 (2018). https://doi.org/10.1002/chem.201705855

[28] K. Wang, Y. Huang, X. Qin, M. Wang, X. Sun, M. Yu, Synthesis of hollow SnO_2/SnS_2 hybrids and their application in sodium-ion batteries. ChemElectroChem **4**(9), 2308-2313 (2017). https://doi.org/10.1002/celc.201700309

[29] X. Zhang, J. Xiang, C. Mu, F. Wen, S. Yuan, J. Zhao, D. Xu, C. Su, Z. Liu, SnS_2 nanoflakes anchored graphene obtained by liquid phase exfoliation and MoS_2 nanosheet composites as lithium and sodium battery anodes. Electrochim. Acta **227**, 203-209 (2017). https://doi.org/10.1016/j.electacta.2017.01.036

[30] S. Zhang, H. Zhao, L. Yue, Z. Wang, J. Mi, Fixed-bed assisted synthesis SnO_2/ SnS_2/CNTs composite for enhanced sodium storage performance. J. Alloys Compd. **717**, 127-135 (2017). https://doi.org/10.1016/j.jallcom.2017.05.055
